# The serum vitamin D status in adults with diabetic retinopathy: A systematic review and meta-analysis

**DOI:** 10.5937/jomb0-60670

**Published:** 2026-01-28

**Authors:** Hongyu Wei, Hao Wang, Ke Diao, Xiaoxuan Wang, Hong Chen, Congying Wang, Minglian Zhang

**Affiliations:** 1 Graduate School of Hebei University of Chinese Medicine, Shijiazhuang, China; 2 Hebei Eye Hospital, The Affiliated Hospital of Hebei University o f Chinese Medicine Xingtai, China; 3 Hebei Eye Hospital, The Affiliated Hospital of Hebei University of Chinese Medicine, Xingtai, China

**Keywords:** diabetic retinopathy, vitamin D, 25-hydroxy-vitamin D3, 1,25-dihydroxyvitamin D3, meta-analysis, dijabetička retinopatija, vitamin D, 25-hidroksivitamin D3, 1,25-dihidroksivitamin D3, meta-analiza

## Abstract

**Background:**

Emerging evidence suggests that vitamin D may play a crucial role in the development of diabetic retinopathy (DR). However, the extent of vitamin D deficiency in individuals with DR remains uncertain. This study aimed to evaluate serum vitamin D levels in adults with DR.

**Methods:**

A literature was retrieved using the Embase, PubMed and Cochrane databases to identify observational studies that evaluated the levels of 25(OH)DS, 1,25(OH)2D5 or total 25(OH)D in the serum of patients with DR. A total of 31 independent studies were included for meta-analysis.

**Results:**

The pooled mean concentration of total 25(OH)D among individuals with diabetes was 16.04 ng/mL (95% CI: 15.13-16.96; I2 = 98.8%), while the mean serum level of 25(OH)D3 in those with DR was 10.68 ng/mL (95% CI: 5.94-15.41; I2 = 99.5%), both significantly below the normal reference range. The average concentration of 1,25(OH)2D3 was 31.14 pg/mL (95% CI: 24.35-37.94; I2 = 98.8%).

**Conclusions:**

The evidence from this meta-analysis indicates an association between vitamin D deficiency and an increased risk of DR.

## Abstract (sr)

**Uvod:**

Novi dokazi ukazuju na to da vitamin D može igrati ključnu ulogu u razvoju dijabetičke retinopatije (DR). Međutim, stepen nedostatka vitamina D kod osoba sa DR ostaje neizvestan. Cilj ove studije bio je da se procene nivoi vitamina D u serumu kod odraslih sa DR.

**Metode:**

Prikupljena je literatura korišćenjem baza podataka Embase, PubMed i Cochrane kako bi se identifikovale opservacione studije koje su procenjivale nivoe 25(OH)D3, 1,25(OH)2D3 ili ukupnog 25(OH)D u serumu pacijenata sa DR. Ukupno 31 nezavisna studija je uključena u meta-analizu.

**Rezultati:**

Zbirna prosečna koncentracija ukupnog 25(OH)D kod osoba sa dijabetesom bila je 16,04 ng/mL (95% CI: 15,13-16,96; I2 = 98,8%), dok je prosečan nivo 25(OH)D3 u serumu kod osoba sa DR bio 10,68 ng/mL (95% CI: 5,94-15,41; I2 = 99,5%), oba značajno ispod normalnog referentnog opsega. Prosečna koncentracija 1,25(OH)2D3 bila je 31,14 pg/mL (95% CI: 24,35-37,94; I2 = 98,8%).

**Zaključak:**

Dokazi iz ove meta-analize ukazuju na vezu između nedostatka vitamina D i povećanog rizika od DR.

## Introduction

DR stands as the most common ocular problems globally and serves as the predominant factor contributing to sight loss among people afflicted with diabetes [1]. Statistical data indicate that roughly a third of the world's populace diagnosed with diabetes will experience DR [2]. The etiology of DR is multifactorial, encompassing long-term hyperglycemia, oxidative stress, inflammatory response, vascular endothelial dysfunction and other links [3]. Vitamin D, a crucial lipid-soluble vitamin and steroid hormone, is vital for regulating calcium and phosphorus metabolism and modulating immune responses in the body [4]. In recent years, a growing collection of studies has centered on the influence of vitamin D in the growth and spread of DR. Research indicates that individuals with DR frequently display diminished amounts of vitamin D, implying that a decrease in vitamin D could potentially accelerate the advancement of DR [5]. Vitamin D is stored in the body primarily as 25(OH)D_3_. This form undergoes a two-step hydroxylation (in the liver and kidneys) to become the physiologically active form, 1,25(OH)_2_D_3_ [6]. Serum 25(OH)D_3_ levels indicate vitamin D nutritional status, while 1,25(OH)_2_D_3_ levels reflect its physiological activity [7]. Studies show that individuals with diabetic retinopathy (DR) have significantly lower serum levels of both 25(OH)D_3_ and 1,25(OH)_2_D_3_ compared to diabetic controls without DR [8] [9]. Furthermore, vitamin D levels are inversely correlated with the severity of DR [10].

The findings from various studies exhibited a lack of complete consistency; for instance, specific studies did not identify an observable and meaningful disparity in vitamin D concentrations among the DR and untreated cohorts [11]. Considering the heterogeneity and limitations of the available evidence, it is necessary to undertake a comprehensive review and quantitative assessment of pertinent studies. In this study, we systematically searched domestic and foreign databases by comprehensive evaluation, and encompassed cohort or case-control investigations that assessed serum 25(OH)D_3_, 1,25(OH)_2_D_3_, or total 25-hydroxyvitamin D concentrations among individuals with DR, quantitatively synthesized the existing evidence, and evaluated the overall vitamin D levels of DR patients, in order to provide reference for clinical practice. At the same time, the heterogeneity and publication bias of the included studies were also assessed to judge the reliability of the results.

## Materials and methods

### Methodology for searching literature

A comprehensive investigation was undertaken utilizing the Cochrane Library, PubMed, and Embase records, covering the period from the start up to September 2024. The search strategy employed involves the integration of subject headings and freetext terms, utilizing the primary keywords comprising: »vitamin D,« »cholecalciferol,« »25-hydroxyvitamin D,« »1,25-dihydroxyvitamin D,« »diabetic retinopathy,« and »diabetes complications«. References to included studies were also retrospectively used to supplement missing studies.

### Criteria for exclusion and inclusion

Inclusion criteria: Studies involving human subjects with DR; reporting serum 25(OH)D_3_, 1,25(OH)_2_D_3_, or Total 25(OH)D levels; diagnosing DR using the ICO clinical grading system [12]; employing case control, cohort, or, cross-sectional designs. Exclusion Criteria: Non-original studies reviews, meetings, case reports; animal or cell studies; duplicate publications; studies lacking extractable key data.

### Data extraction and literature assessment

Two scholars conducted an independent examination of the works in question. The extracted information mainly encompassed: name of the initial writer, the year of release, the field of investigation, the sex and age of the subjects involved in the research, the description and grading of diabetes and DR, the detection method, the average and standard deviation of serum vitamin D concentrations within every cohort, along with the modification of confounding factors. Two peer reviews meticulously verified the inclusion outcomes and extracted data, engaging an additional reviewer to address any discrepancies that arose.

### Quality evaluation adoption

The Newcastle-Ottawa Scale evaluates the efficacy of case-control investigations [13]. The scale is scored from three aspects: choice (4 points), comparability (2 points), and exposure (3 points), aggregating to 9 points. A score of ≥ 7 is of high quality, 5-6 is of moderate quality, and < 5 is of low quality.

### Data analysis

A thorough evaluation was carried out utilizing Stata software. The effect size of each study was assessed through serum 25(OH)D_3_, 1,25(OH)_2_D_3_, or total 25(OH), utilizing the mean of D levels along with its 95% Cl. The I^2^ test was employed to assess heterogeneity among the investigations, revealing that an I^2^ value exceeding 50% indicated considerable variability. Once the effects were found to be heterogeneous (I^2^ > 50%), a random effects model was used. Otherwise, a fixed effect model was used. The risk of publishing bias of the featured investigations evaluated visually by funnel plots. To evaluate the robustness of the findings, a sensitivity analysis was carried out, which also explored the effect of excluding one article at a time on the overall risk calculation. Statistical significance was set at P < 0.05.

## Results

### Outcomes of the search

**Figure 1:**
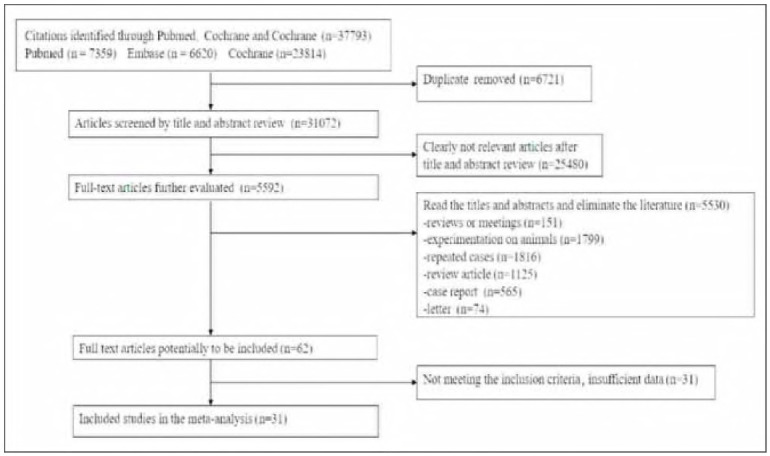
Document retrieval flow chart.

The initial searches revealed a total of 37,793 papers within the Embase, PubMed, and Cochrane databases, comprising 7,359 from PubMed, 6,620 from Embase, and 23,814 from Cochrane. Following the removal of 6721 duplicate publications, 31,072 papers were evaluated through their titles and abstracts, leading to the marginalization of 25,480 distinctly irrelevant works, and 5592 articles underwent full-text evaluation. In the full-text screening process, 151 reviews or conference proceedings, 1799 animal experimental studies, 1816 duplicate cases, 1125 review articles, 565 case reports, and 74 correspondences were excluded. Finally, 62 articles were included for full-text reading and data extraction. Upon thorough examination of the complete text, 31 investigations that failed to satisfy the eligibility standards or lacked adequate data were subsequently excluded, and 31 studies were finally included for quantitative synthesis and meta-analysis (Figure 1). 12,227 people with diabetes were included in all included studies. Among them, 5256 males and 5887 females were explicitly reported, and the rest of the studies did not report gender composition. The included studies were geographically wide, including China, India, the Middle East, Europe, and the United States (Table 1) 14-44.

**Table 1:** Characteristics of observational studies included in this meta-analysis.

Study	Country/<br>Region	Year	Subjects<br>(male/female)	Mean age	Type of<br>disease	Total 25<br>(OH)D	1,25(OH)2D3		25(OH)D3	
Ahmed et<br>al. [14]	Qatar	2021	460<br>(227/233)	55.2±15.0	T2DM	Total 25<br>(OH)D	26.46±15.30<br>ng/dL			
Ahmed et<br>al. [15]	Qatar	2020	274<br>(110/164)	55.2±9.9	T2DM					25(OH)D_3_
Almoosa et<br>al. [16]	Bahrain	2019	300<br>(NR)	NR	T2DM	Total 25<br>(OH)D	12.6±7.4<br>ng/mL			
Bajaj et al.<br>[10]	India	2014	158<br>(95/63)	52.85±8.26	T2DM	Total 25<br>(OH)D	19.25± 7.86<br>ng/mL			
Butler et al.<br>[17]	Qatar	2020	460<br>(NR)	56.0±10.4	T2DM	Total 25<br>(OH)D	6.49±7.53<br>ng/mL	1,25(OH)_2_D_3_	0.025±0.023<br>ng/mL	25(OH)D_3_
Castillo-Oti<br>et al. [18]	Spanish	2022	385<br>(213/172)	69.89±9.86	T2DM	Total 25<br>(OH)D	15.50±10.80<br>ng/mL	1,25(OH)_2_D_3_	24.50±13.00<br>pg/mL	
Choudhary<br>et al. [19]	India	2024	119<br>(67/52)	49.5±11	T2DM	Total 25<br>(OH)D	16.3±9.2<br>ng/mL	1,25(OH)_2_D_3_	42.5±15.6<br>pg/mL	
Hassan et<br>al. [20]	Saudi<br>Arabia	2023	252<br>(114/138)	18-87	T2DM	Total 25<br>(OH)D	15.50±7.60<br>ng/mL	1,25(OH)_2_D_3_	37.40±9.20<br>pg/mL	
He et al.<br>[21]	China	2014	1520<br>(773/747)	59.03±11.67	T2DM	Total 25<br>(OH)D	15.36±4.81<br>ng/mL			
Jung et al.<br>[22]	South<br>Korea	2016	257<br>(111/146)	58.8±12.1	T2DM	Total 25<br>(OH)D	14.4±8.6<br>ng/mL			
Wan et al.<br>[23]	China	2019	4767<br>(2050/2717)	67±9	T2DM	Total 25<br>(OH)D	16.34± 5.67<br>ng/mL			
Lopes et al.<br>[24]	Portugal	2020	182<br>(86/96)	43±14	T1 DM	Total 25<br>(OH)D	20.3±10.7<br>ng/mL			
Nadri et al. <br>[25]	India	2021	88<br>(52/36)	53.18	T2DM	Total 25<br>(OH)D	14.07±1.21<br>ng/mL			
Nadri et al.<br>[26]	India	2019	72<br>(NR)	53	T2DM	Total 25<br>(OH)D	14.10±1.20<br>ng/mL			
Navaei et al.<br>[27]	Iran	2023	278<br>(93/185)	62.2±4.46	T2DM	Total 25<br>(OH)D	25.4±12.6<br>ng/mL			
Payne et al.<br>[8]	United<br>States	2012	42<br>(21/21)	59.8±12.0	T2DM	Total 25<br>(OH)D	21.1 ±10.5<br>ng/mL			
Reddy et al.<br>[28]	India	2015	82<br>(48/34)	57.5±9.3	T2DM	Total 25<br>(OH)D	16.9±7.2<br>ng/mL			
Reheem et<br>al. [29]	Egypt	2013	200<br>(96/104)	69±2.6	T2DM	Total 25<br>(OH)D	31.6±12.01<br>nmol/L	1,25(OH)_2_D_3_	34.1 ±17.2<br>pmol/L	
Saxena et<br>al. [30]	India	2019	66<br>(NR)	40-65	T2DM	Total 25<br>(OH)D	14.07±1.21<br>ng/mL			
Senyigit et<br>al. [31]	Turkey	2019	163<br>(NR)	56.69±7.94	T2DM					25(OH)D_3_
Seyyar et al.<br>[32]	Turkey	2022	165<br>(73/92)	62.5±6.8	T2DM	Total 25<br>(OH)D	11.77±7.86<br>ng/mL			
Seyyar et al.<br>[33]	Turkey	2022	178<br>(87/91)	61.5 ± 11.5	T2DM	Total 25<br>(OH)D	10.8±4.47<br>ng/mL			
Shimo et al.<br>[34]	Japan	2014	75<br>(28/47)	28.5±5.5	T1 DM					25(OH)D_3_
Usalp et al.<br>[35]	Turkey	2023	40<br>(15/25)	58.9±7.8	T2DM	Total 25<br>(OH)D	12.8±4.2<br>ng/mL			
Usluogullari <br>et al. [36]	Turkey	2015	557<br>(296/261)	55.2±10.9	T2DM	Total 25<br>(OH)D	19.1 ±8.1<br>ng/mL			
Vaghela et<br>al. [37]	India	2022	98<br>(57/43)	58.9±13.6	T2DM	Total 25<br>(OH)D	14.4± 5.9<br>ng/mL			
Verma et al.<br>[38]	India	2024	98<br>(52/46)	57.6±10.4	T2DM	Total 25<br>(OH)D	25.91 ±7.83<br>ng/mL			
Yuan et al.<br>[39]	China	2019	273<br>(143/130)	58.10±10.83	T2DM	Total 25<br>(OH)D	13.14±8.71<br>ng/mL			
Zahedi et al.<br>[40]	Iran	2024	201<br>(134/68)	60.0±8.1	T2DM	Total 25<br>(OH)D	14.46±7.88<br>ng/mL			
Zhao et al.<br>[41]	China	2021	235<br>(120/89)	61 ±6.8	T2DM	Total 25<br>(OH)D	16.38±9.16<br>ng/mL			
Zhuang et<br>al. [42]	China	2024	182<br>(95/87)	51.1 ±7.6	T2DM	Total 25<br>(OH)D	17.34±4.40<br>ng/mL			

### Quality evaluation

The NOS score of the investigations included in this analysis varied between 5 and 7, suggesting that the general standard of these investigations was considered satisfactory.

### The meta-analysis of serum 25 (OH) D_3_ level

**Figure 2:**
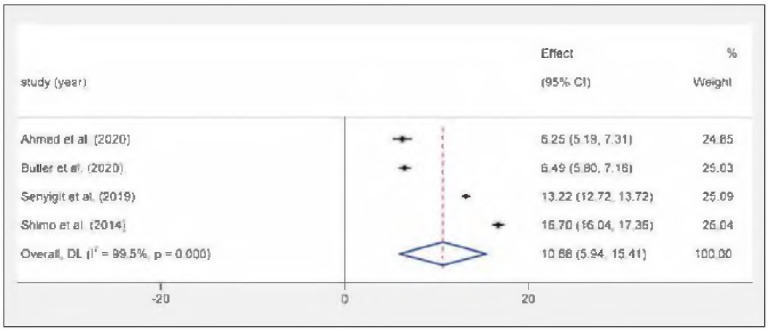
Forest map of serum 25(OH)D_3_ levels in diabetic retinopathy patients.

This review synthesized four independent studies to examine the serum 25(OH)Ds levels in persons experiencing DR. Given the clear variability in the results (I^2^ = 99.5%, P = 0.000), a random-effects model was utilized for the combined evaluation. The mean of 25(OH)D_3_ in the four studies was 10.68 ng/mL (95% Cl: 5.94-15.41) after pooling. The particular outcomes are illustrated in Figure 2. To gauge the impact of particular investigations on the outcomes of the systematic review, we further performed sensitivity analyses. The consistency of the comprehensive meta-analysis findings can be assessed by sequentially excluding individual studies and comparing the alterations in pooled effects and their confidence intervals before and after each exclusion. Sensitivity analysis showed that there were no obvious changes in the pooled mean value as a result of the exclusion of any other single study.

### The meta-analysis of serum 1,25(OH)_2_D_3_

**Figure 3:**
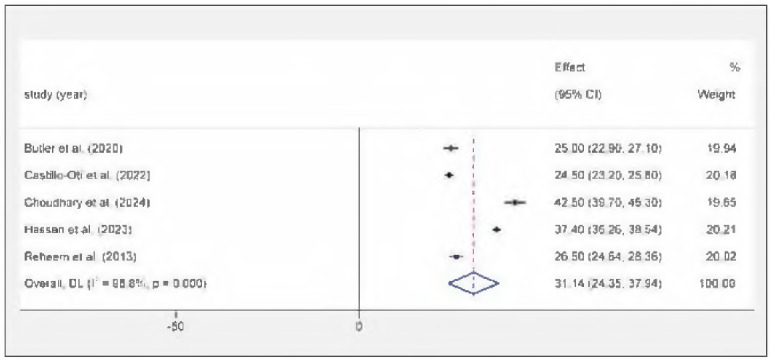
Forest map of serum 1,25(OH)_2_D_3_ levels in diabetic retinopathy patients.

This study incorporated five independent investigations, and Figure 3 illustrates the outcomes of a random-effects meta-analysis of serum 1,25(OH)_2_D_3_ amounts in persons experiencing DR. The results exhibited considerable variability (I^2^ = 98.8%, P<0.001). The combined 1,25(OH)_2_D_3_ mean of the five studies was 51.14 pg/mL (95% Cl: 24.35-37.94). The specific results are shown in Figure 3. We undertook sensitivity evaluations to assess the influence of particular investigations on the aggregate effect size of 1,25(OH)_2_D_3_ levels. The robustness of the results was investigated by excluding the included studies one by one and observing changes in the meta-analysis, projections of effect size. Sensitivity analysis showed that there were no obvious changes in the pooled mean value as a result of the exclusion of any other single study.

### The meta-analysis of serum total 25(OH)D levels

**Figure 4:**
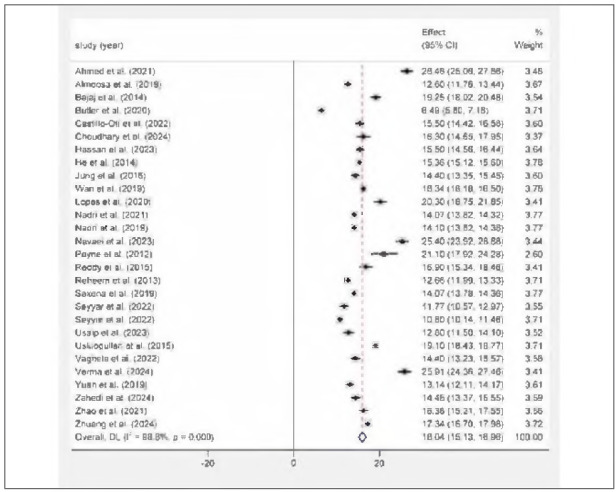
Forest map of serum Total 25(OH)D levels in diabetic retinopathy patients.

This meta-analysis included twenty-eight studies on serum total 25(OH)D amounts in those diagnosed with T2DM, conducted between 2012 to 2024. The mean serum Total 25(OH)D concentrations in individuals diagnosed with T2DM was 16.04 ng/mL (95% Cl: 15.13-16.96 ng/mL) employing a randomeffects model evaluation. The average total 25(OH)D amounts observed in the investigations varied between 6.49 ng/mL and 26.46 ng/mL. The investigations exhibited considerable variability (l^2^ = 98.8%, P<0.001). Notably, most studies reported mean concentrations of 25(OH)D under 20 ng/mL indicate that deficiencies in vitamin D could be prevalent among individuals with T2DM. This discovery holds significant relevance for the medical management of diabetes. The specific results are depicted in Figure 4. We carried out sensitivity evaluations to gauge the influence of particular investigations on the overall effect size of Total 25(OH)D levels. The 28 included studies were sequentially excluded and alterations in the effect size estimates from the meta-analysis were noted to assess the reliability of the findings. Sensitivity analysis showed that there were no obvious changes in the pooled mean value as a result of the exclusion of any other single study.

### Publication bias assessment

**Figure 5:**
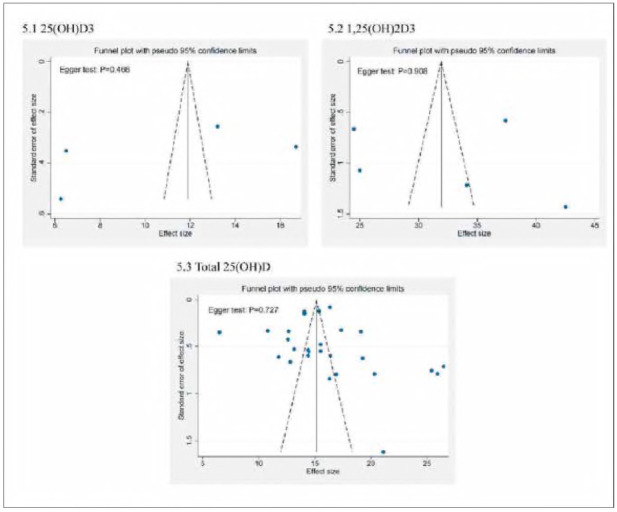
Serum 25(OH)D_3_, 1,25(OH)_2_D_3_ and Total 25(OH)D funnel plot.

We assessed the likelihood of publication bias in the investigations that were included through plotting funnel plots for serum 25(OH)D_3_, 1,25(OH)_2_D_3_, and Total 25(OH)D (Figure 5). Visually, the funnel plot's left and right sides are nearly symmetrical. In order to quantitatively evaluate the extent of bias in publications, we conducted Egger's linear regression test for each of the three indicators. The results showed that the P values of Egger's test for 25(OH)D_3_, 1,25(OH)_2_D_3_ and Total 25(OH)D were 0.466, 0.908 and 0.727, respectively, which were greater than the significance threshold of 0.05. This indicates that no obvious publication bias was observed in this metaanalysis.

## Discussion

This study employed a meta-analysis and systematic review to measure the evidence regarding vitamin D levels among individuals suffering from DR. The findings indicated that serum amounts of 25(OH)D_3_, 1,25(OH)_2_D_3_, and Total 25(OH)D in persons with DR were lower, implying an intriguing link among insufficient amounts of vitamin D and the initiation and advancement of DR, which aligns with some previous studies [5] [6] [10]. The findings were broadly consistent, but the results were more convincing as more high-quality studies were included in this study.

DR is a prevalent microvascular problem associated with diabetes, and its development is influenced by a range of factors [43] [44]. Vitamin D plays part in the pathogenic course of DR through various processes. First, vitamin D plays a role in inhibiting the inflammatory response and reducing the production of cytokines that are pro-inflammatory, thus reducing inflammatory damage to retinal micro vessels. Secondly, vitamin D has an antioxidant effect, which can remove excess reactive oxygen species (ROS) and safeguard retinal pigment epithelial cells and ganglion cells [45]. Thirdly, vitamin D can enhance the synthesis of anti-vascular endothelial growth factor and suppress pathological neovascularization [46]. Moreover, vitamin D enhances the function of vascular smooth muscle cells and mitigates microcirculation disorders via management of calcium ion influx [47]. Considering the various protective benefits associated with vitamin D, correcting vitamin D insufficiency is expected to impede the advancement of DR. The results of this study showed that vitamin levels in patients with DR were significantly lower than those in patients with non-diabetic retinopathy. This suggests that retinopathy in diabetic patients may be closely related to vitamin D deficiency and that our findings provide strong evidence for guiding the clinical management of diabetic patients, thereby helping to reduce retinopathy.

The findings of the systematic review conducted in this study indicated a notable level of heterogeneity among the investigations that were included, with I^2^ values exceeding 98%. Heterogeneity can stem from a variety of factors. First, participants in the studies included exhibited variations in baseline characteristics, including age, gender, ethnicity, duration of diabetes, and comorbidities. These confounding factors may influence vitamin D quantities and bring about discrepancies in effect sizes across studies [48]. Secondly, the diagnostic criteria for DR in each study were different, some studies used fundus photography, some used fluorescein fundus angiography, and some studies did not provide a clear basis for diagnosis, and the heterogeneity of DR diagnosis directly led to differences in patient characteristics [49]. Thirdly, significant differences exist in the detection methods for serum 25(OH)D_3_, 1,25(OH)_2_D_3_, and Total 25(OH)D. The techniques encompass liquid chromatography-mass spectrometry, radioimmunoassay, and chemiluminescence immunoassay (CLIA). Performance indicators such as sensitivity, specificity, and detection limits vary considerably among these methods, potentially resulting in systematic errors in vitamin D level assessments [50]. In addition, the geographical distribution of the included studies is wide, covering different countries and places like China, India, the Middle East, Europe, and United States, and variations in race, eating habits, and sunshine hours could influence the manufacturing and breakdown of vitamin D in the human body [51]. Subgroup analysis indicated that levels of 25(OH)D_3_ and Total 25(OH)D were elevated in European and American populations compared to the Asian population (P<0.05), highlighting the significance of ethnic and regional differences as sources of heterogeneity^2^. Finally, the sample sizes of the investigations included in the analysis exhibited considerable variability, with smaller studies demonstrating a greater propensity to yield extreme outcomes, resulting in a deviation from the true effect size estimates [52]. Considering the above factors, the high heterogeneity reduces the comparability and robustness of the results of this study to a certain extent.

This study has certain limitations. The studies included were all single arm, making it challenging to ascertain the link of causation between the quantities of vitamin D and DR towing to the absence of appropriate control groups. Reduced quantities of vitamin D may be a consequence of DR, or both could be related to other factors. Prospective cohort studies can help to further clarify the relationship between the two. Second, funnel plots suggest that there may be publication bias, and negative studies with small sample sizes may be difficult to publish, resulting in results that deviate from the true effect. Finally, the quality of individual studies varied and may be affected by selection bias, information bias, and confounding bias. We assessed only the overall quality of the studies during the inclusion process, lacking a more detailed assessment of the danger of bias. The limitations may have influenced the dependability of the investigation's findings. From the above limitations, future research directions include: (1) conducting cohort studies with large samples and extensive follow-up to assess the correlation between low levels of vitamin D and the risk of DR; (2) to explore the differences of vitamin D in patients with different stages and types of DR, along with the dose-response connection between vitamin D and the seriousness of DR; (5) carry out clinical intervention investigations into vitamin D supplemental intake to evaluate the impact of vitamin D therapy on the advancement of DR; Comprehensive examination of the molecular mechanism connected to low-level vitamin D promoting the existence and growth of DR. These studies elucidate the significance of vitamin D in both the avoidance and control of DR and provide innovative ideas for the development of intervention strategies.

## Conclusion

In conclusion, the study demonstrates that a lack of vitamin D is often found in individuals with DR, implying that vitamin D may be involved in DR development. Given the prominent level of heterogeneity and potential for publication bias, more high-quality prospective investigations are required to substantiate this conclusion. In future studies, it is essential to assess the link between reduced amounts of vitamin D and prognosis of DR, along with exploring the potential clinical utilization of vitamin D in both the avoidance and control of DR.

## Dodatak

### Funding

This work was supported by the Optimization of Integrated Traditional Chinese and Western Medicine Diagnosis and Treatment Plan for Retinal Artery Occlusion and Construction of Intelligent Auxiliary Diagnosis and Treatment System (N0.23377712D); The High-level Key Discipline Construction Project of the National Administration of Traditional Chinese Medicine (zyyzdxk-2023017); Xingtai City Innovation Capacity Enhancement Program Project (No. 2023ZZ106); Innovation Support Project for Post-graduates in Hebei Province (XCXZZBS2025026).

### Conflict of interest statement

All the authors declare that they have no conflict of interest in this work.
